# Ferroptosis-Related Gene Signatures: Prognostic Role in HPV-Positive Oropharyngeal Squamous Cell Carcinoma

**DOI:** 10.3390/cancers17030530

**Published:** 2025-02-05

**Authors:** Deborah Lenoci, Mara Serena Serafini, Marta Lucchetta, Stefano Cavalieri, Ruud H. Brakenhoff, Frank Hoebers, Kathrin Scheckenbach, Tito Poli, Lisa Licitra, Loris De Cecco

**Affiliations:** 1 Integrated Biology of Rare Tumors, Department of Experimental Oncology, Fondazione IRCCS Istituto Nazionale Dei Tumori, Via Venezian 1, 20133 Milan, Italy; deborah.lenoci@istitutotumori.mi.it (D.L.); mss4008@med.cornell.edu (M.S.S.); marta.lucchetta@istitutotumori.mi.it (M.L.); 2 Head and Neck Medical Oncology Department, Fondazione IRCCS Istituto Nazionale dei Tumori di Milano, 20133 Milan, Italy; stefano.cavalieri@istitutotumori.mi.it (S.C.); lisa.licitra@istitutotumori.mi.it (L.L.); 3 Department of Oncology and Hemato-Oncology, University of Milan, 20122 Milan, Italy; 4 Department of Otolaryngology-Head and Neck Surgery, Amsterdam UMC Location Vrije Universiteit Amsterdam, De Boelelaan 1117, 1081 HV Amsterdam, The Netherlands; rh.brakenhoff@amsterdamumc.nl; 5 Cancer Biology and Immunology, Cancer Center Amsterdam (CCA), 1081 HV Amsterdam, The Netherlands; 6 Department of Radiation Oncology (MAASTRO), Research Institute GROW, Maastricht University, 6229 ET Maastricht, The Netherlands; frank.hoebers@maastro.nl; 7 Department of Otolaryngology, Medical Faculty, Heinrich Heine University Düsseldorf, 40225 Düsseldorf, Germany; scheckenbach@med.uni-duesseldorf.de; 8 Unit of Maxillofacial Surgery, Department of Medicine and Surgery, University of Parma-University Hospital of Parma, 43126 Parma, Italy; tito.poli@unipr.it

**Keywords:** oropharynx squamous cell carcinoma, HPV-positive, ferroptosis signatures, prognosis, biomarker, immune microenvironment

## Abstract

Background: Despite advances in the management of head and neck squamous cell carcinoma (HNSCC), prognostic models and treatment strategies remain inadequate, particularly for HPV-positive oropharyngeal squamous cell carcinoma (OPSCC). The rising incidence of HPV-positive OPSCC highlights an urgent need for innovative therapeutic approaches. Ferroptosis, a regulated form of non-apoptotic cell death, has gained attention for its role in cancer progression, but its potential as a prognostic and therapeutic target in HPV-positive OPSCC remains largely unexplored. This study investigates the role of ferroptosis in HPV-positive OPSCC, aiming to identify prognostic markers and provide insights into potential therapeutic strategies that could improve patient outcomes. Methods: Thirteen ferroptosis gene expression signatures were retrieved from the literature, and their performance and association to the immune microenvironment were validated on a meta-analysis of 267 HPV-positive cases (Metanalysis-HPV267) and 286 samples from the BD2Decide project (BD2-HPV286). Results: Our analysis revealed that specific ferroptosis-related gene expression signatures, particularly FER3, FER4, FER6, and FER12, are significantly associated (*p*-value < 0.05) with high-risk patient groups and adverse tumor microenvironment features, including suppressed immune activity and enhanced stromal involvement. Elevated expression of CAV1, a ferroptosis suppressor, further delineates high-risk profiles. Conclusions: These findings highlight the prognostic significance of ferroptosis in stratifying patients and identifying those with poorer clinical outcomes. Targeting ferroptosis pathways represents a novel and promising approach to addressing the unmet need for effective prognostic and therapeutic strategies in HPV-positive OPSCC. Future research should focus on translating these findings into clinical applications to advance precision oncology and improve outcomes for this growing patient population.

## Simple Summary

Biomarkers to improve high-precision treatment for HPV-positive oropharyngeal squamous cell carcinoma (OPSCC) patients are urgently awaited. We have previously studied the biological and immune characteristics of HPV-positive OPSCC, classifying patients into three risk groups: low (Cl1), intermediate (Cl3), and high (Cl2). Ferroptosis, a non-apoptotic form of cell death, has been observed in various cancers, including head and neck squamous cell carcinoma (HNSCC). This suggests that ferroptosis could be important in understanding prognosis and the tumor microenvironment in OPSCC. We aimed to evaluate the prognostic value of ferroptosis-related gene signatures. These signatures were first identified and validated using data from The Cancer Genome Atlas (TCGA) head and neck squamous cell carcinoma (HNSCC) dataset. To further confirm their potential, we tested these signatures in additional independent datasets and the BD2 cohort, including a meta-analysis.

## 1. Introduction

Head and neck squamous cell carcinoma (HNSCC) is the sixth most common cancer, and according to GLOBOCAN estimates on HNSCC, 890,000 cases and 450,000 deaths were reported worldwide [1]. HNSCC is an extremely heterogeneous disease, despite that most of the cases occur in the mucosa of the oral cavity, oropharynx, hypopharynx, and larynx [1]. Tobacco, alcohol abuse, betel nuts, and viral infection are the main etiological risk factors. In recent years the incidence of tobacco-related HNSCC is decreasing, due to the increased awareness of risk factors and improved self-care [2], but the incidence of HNSCC due to human papillomavirus (HPV) infection, mainly presenting as oropharyngeal cancer (OPSCC), is gradually increasing [3] and is expected to grow further in the next decades despite the availability of HPV vaccination [4]. The disease harboring from HPV infection has a peculiar genesis, and the 8th edition of AJCC TNM staging system defined HPV-pos OPSCC using surrogate marker p16 as a clinical entity [5]. A favorable clinical outcome is frequently associated with HPV-pos OPSCC, and according to cancer cases prediction, the number of HPV-pos OPSCC is expected to keep increasing between 2020 and 2040 [6]. Despite the better prognosis associated with HPV-related diseases, OPSCC patients are often treated with a “one-size-fits-all” approach, regardless of their p16/HPV status. While de-intensified treatment strategies have been explored, many have failed, likely due to the lack of a robust patient selection process [7]. Translational research provides a valuable opportunity to categorize patients into distinct prognostic groups based on their molecular and clinical profiles but treated by uniform protocols. Gene expression data, in particular, shows great potential in this regard. We previously demonstrated the heterogeneity of HPV-related OPSCC by subtyping the disease into three histologically and clinically distinct clusters, each with its own prognosis [8]. Given this, further investigation into the complex biology of HPV-related OPSCC is crucial for improving our understanding and, ultimately, enhancing treatment options for patients, particularly for the low prognostic groups. In this context, further dissecting the complexity of HPV-related OPSCC biology is a crucial need to improve the knowledge and hopefully the treatment options at the patient’s bedside.

One of these opportunities is provided by the study of cell death. More than two decades ago, the cancer hallmarks were highlighted [9]. Uncontrolled growth and abnormal cell proliferation were marked as essential features; however, it was clear that they were not sufficient for cancer to progress. Among all, the evasion of programmed cell death (i.e., resistance to apoptosis) was underlined as a highly contributing mechanism in cancer genesis and establishment. Historically, cell death was divided into three forms based on cell morphological features: apoptosis, autophagy, and necrosis [10]. Apoptosis is the best understood and the most extensively studied programmed cell death mechanism. The manifestation of apoptosis includes distinct changes, such as nuclear fragmentation and mRNA decay [11,12]. Autophagy is defined as a preserved catabolic degradation, where damaged organelles, pathogens, and cytoplasmic macromolecules are sent inside lysosomes for digestion [13]. In contrast to apoptosis, dying necrotic cells display increased cell volume, swelling of organelles, and loss of membrane integrity [14].

Advances in basic research allowed for the characterization of new cell death types, and in 2012, a form of regulated cell death, named “ferroptosis”, was identified by Dixon et al. [10]. Biologically speaking, ferroptosis per se is not exactly a form of cellular death, but more a mechanism of cellular self-harm that can lead to death; nevertheless, it meets the criteria for being in an instance classified as a form of regulated cell death [15].

Iron is an essential element in mammal cells, and it plays an important role in different biological and metabolic processes, such as DNA synthesis and oxygen transportation [16]. Under normal conditions, iron balance is maintained with the physiological function of the cell; however, when the cellular iron balance is disrupted, it can lead to intracellular iron overload [17].

Ferroptosis was originally discovered in an oncogenic Ras-expressing human foreskin fibroblast cell line by small compounds against RAS mutation (such as erastin and RSL3), today considered as ferroptosis-inducing agents. The process of ferroptosis is initiated by specific perturbations of intracellular microenvironments, such as severe lipid peroxidation, relying on reactive oxygen species (ROS) generation and iron availability [18]. These molecular abnormalities resulted from the loss of selective permeability of the plasma membrane. Therefore, cells undergoing ferroptosis display distinct cellular morphologies, including decreased density, volume shrinkage, outer mitochondrial membrane rapture, and decreased or vanished mitochondrial cristae [12,19]. Ferroptosis may be induced by a variety of physiological and pathological processes in humans, and recently research revealed its strong involvement in human disease. In particular, ferroptosis plays an important role in the occurrence and progression of various cancer types, such as breast cancer [20], lung cancer [21], and head and neck cancer [22,23].

Because iron is an essential element of tumor cells, their proliferation is usually more dependent on iron than normal cells. As a result, tumor cells are more sensitive to damage associated with iron excess. Consequently, ferroptosis plays a fundamental role also in enhancing treatment response [24]. As an example, it has been observed that radiotherapy can damage DNA and increase oxidative stress, inevitably inducing ferroptosis [25]. Moreover, emerging evidence suggested the potential role of ferroptosis in tumor suppression and immune regulation and enhancement, increasing tumor cell sensitivity to drugs [25,26,27,28,29]. Thus, ferroptosis is a potential future panacea for a variety of cancers, and its induction can significantly inhibit tumor development and improve patient prognosis [30]. To note, several lines of evidence indicated that ferroptosis could be triggered by viral infection [31], and Epstein–Barr virus (EBV) and Hepatitis B Virus (HBV) [32,33] are two examples of pathogens modulating the ferroptosis pathway. Through viral latency programs, for example, EBV infects and converts B cells into immortalized lymphoblastoid cells, increasing ROS levels and ferroptosis vulnerability during transformation stages [34]. Recent studies demonstrated the correlation of ferroptosis and iron metabolism in nasopharyngeal carcinogenesis, a well-characterized tumor for which the major risk factor is the EBV infection [35]. Moreover, ferroptosis is under investigation in cervical squamous cell carcinoma with HPV infection, and several studies demonstrated their strict relationship [36,37,38]. Consequently, ferroptosis might be associated with HPV viral infection in HNSCC since it is known that HPV influences lipid metabolism, it can induce chronic oxidative stress by promoting the production of ROS [39], and the HPV-positive cells use more mitochondrial respiration than HPV-negative cells [40]. Since this knowledge, the aim of the present study is to gain an in-depth understanding of the molecular mechanism of ferroptosis in HPV-positive OPSCC and to provide a new extensive comprehension, potentially being ferroptosis an important new drug target, moving forward to reach a personalized medicine approach in the context of HPV-positive HNSCC.

## 2. Materials and Methods

### 2.1. Data Sources

#### 2.1.1. Ferroptosis Gene Expression Signature Eligibility Criteria

A literature survey (source: Google Scholar and PubMed) of ferroptosis signatures published till July 2024 was carried out to evaluate the biological role of this mechanism in HPV-pos HNSCC. The inclusion criteria were as follows: (i) signatures with reported gene identifiers; (ii) details on the bioinformatic procedures; (iii) availability of the gene-specific weight to impute the signature score. We identified thirteen ferroptosis gene expression signatures, all developed on the HNSCC TCGA dataset. Therefore, 13 ferroptosis signatures were selected, and their main characteristics are reported in Supplementary Table S1.

#### 2.1.2. HPV-Positive Gene Expression Dataset Selection

Ten eligible published datasets reporting gene expression data were selected based on the following inclusion criteria: (i) gene expression profiling on primary squamous cell carcinoma lesions; (ii) reported HPV positive status; (iii) availability of overall survival data; (iv) availability of minimal clinical annotations (i.e., age, gender, anatomical subsite, stage, smoking) in at least 3 out of 5 variables per case with <5% of missing allowed values per variable; (v) MIAME (Minimum Information about a Microarray Experiment) compliant data (for each study the main information was reported in Supplementary Table S2) detailing their platforms, providers, assays, technologies, repositories, tumor sample sizes, and HPV detection methods. A total of 11 datasets are listed, with platforms such as Agilent, Illumina, and Affymetrix used for microarray and RNA sequencing technologies. Tumor sample sizes range from 8 to 72 across repositories like GEO, ArrayExpress, and MIAME-VICE. HPV detection methods include p16 immunohistochemistry (IHC), in situ hybridization, DNA qPCR, Sanger sequencing, and genotyping assays targeting E6 and E7 viral genes. These datasets provide diverse resources for analyzing HPV status and its implications in cancer research.

Patients with locoregionally advanced head and neck squamous cell carcinoma (HNSCC, stages III-IV, TNM 7th edition) treated with curative intent were included in the Big Data and Models for Personalized Head and Neck Cancer Decision Support (BD2Decide, NCT028322102) project. BD2Decide is a collaboration of 11 European partners, including five clinical centers, aimed at creating a clinico-genomic database for head and neck cancer. Approved by institutional review boards and ethics committees in March 2016, the study enrolled 1537 patients diagnosed and treated between 2008 and 2017, with follow-up completed in September 2019 [41]. This work focuses specifically on patients with HPV-positive oropharyngeal cancer (OPC). HPV status in OPC samples was determined using p16 immunohistochemistry (IHC), with further testing for p16-positive cases. HPV DNA was assessed using GP5+/6+ PCR targeting 14 high-risk HPV types. Negative cases underwent further testing with HPV16 E7 primers to exclude L1 integration. Alternatively, in situ hybridization using the INFORM HPV III Family 16 Probe targeted 10 high-risk genotypes, and RNA scope assessed E6/E7 mRNA expression for 18 high-risk genotypes. These methods ensured robust HPV testing and characterization in the BD2Decide study population [8].

#### 2.1.3. HPV-Positive Gene Expression Dataset Processing and Integration

A new INT proprietary cohort was collected, including HPV-positive OPSCC cases. Gene expression profiling was conducted using formalin-fixed paraffin-embedded (FFPE) samples obtained through direct manual dissection of tumor tissue slices. The selected specimens contained at least 75% tumor cell content, with no necrosis or surrounding normal tissue. Total RNA was extracted using the miRNeasy FFPE Kit (Qiagen, Valencia, CA, USA), with the process automated on a QIAcube Robotic workstation. For gene expression profiling, 200 ng of total RNA was analyzed using the Human WG-DASL (cDNA-mediated annealing, selection, extension, and ligation) assay with Human HT12 v4.0 BeadChips (Illumina Inc., San Diego, CA, USA), which enables the detection of 29,377 transcripts. All steps (i.e., reverse transcription, oligonucleotide annealing, ligation, amplification, labeling, probe purification, hybridization, and chip washing) were carried out according to the manufacturer’s guidelines. The microarray chips were scanned using an Illumina IScan Reader. The microarray data are MIAME-compliant; the raw data have been submitted to the NCBI Gene Expression Omnibus (GEO) database under accession number GSE262372, and the relevant data were reported in Supplementary Table S2. The gene expression data from the 10 public datasets and our proprietary cohort were integrated following a meta-analysis approach after gene reannotation based on EntrezID. To account for technical variability, batch effect correction was performed using ComBat [42]. The resulting meta-analysis dataset contained 267 HPV-positive HNSCC cases (hereafter “Metanalysis-HPV267”) and 8077 unique coding genes. After clinical data harmonization, 190 cases out of 267 are HPV-related OPSCC. The HNSCC-TCGA (The Cancer Genome Atlas) dataset was excluded, being utilized as a training set for all the retrieved thirteen ferroptosis signatures.

**Figure 1:**
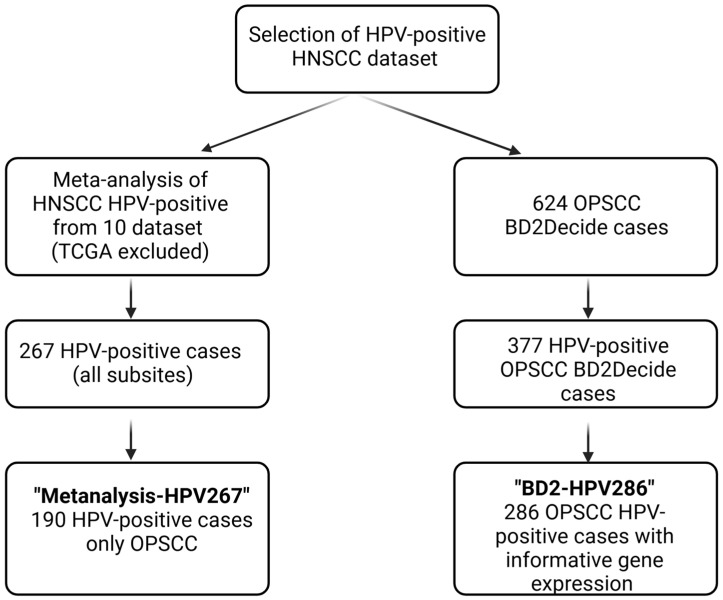
Consort diagram. Selection of HPV-positive HNSCC dataset.

In the H2020 project BD2Decide, 624 OPSCC were enrolled. Gene expression generated using Affymetrix human Clariom D microarrays (Affymetrix, Santa Clara, CA, USA) was informative for 286 OPSCC p16-positive cases, constituting the final “BD2-HPV286” cohort, including 26,904 unique genes [8]. MIAME-compliant data were deposited in the GEO repository (GSE163173). The Metanalysis-HPV267 and BD2-HPV286 cohorts share 7765 coding genes that were used for the subsequent analyses. The workflow of the cohort selection is summarized in Figure 1.

### 2.2. Bioinformatic Analyses of Selected Ferroptosis Signatures

#### 2.2.1. Gene-Expression Signatures

The 13 selected signatures included a total of 57 genes (Table 1). Since we integrated several datasets processed with different platforms, we retained only the common genes; therefore, 15 genes included in the signatures (AURKA, CISD2, FTH1, LINC00336, MAP1LC3A, SOCS1, ZFP69B, SELENOS, SCO2, AKR1C2, CDO1, CYBB, NOX1, NOX3, PROM2) were lost, and the remaining 42 genes were informative for the subsequent analyses.

The role of the genes in the ferroptosis signatures was annotated using a database of ferroptosis regulators, FerrDB (http://www.zhounan.org/ferrdb/current/; accessed on 10 October 2024) [43]. To visualize the number of genes in common among the signatures, we used the UpSetR R package (version 1.4.0) [44].

**Table 1:** Genes considered for the testing of the 13 signatures. Genes in bold are not in the expression matrices.

Signature ID	#Genes	GENES
FRG_1_FHe [45]	7	KEAP1, CDKN2A, EIF2S1, ***FTH1***, MAP3K5, ***SELENOS***, SKC2A3
FRG_2_HLi [46]	10	ATG5, BID, ACO1, GOT1, AKR1C3, GLS2, AKR1C3, ALOX15, ***SCO2***, MAP1LC3A, *MAP3K5*
FRG_3_SLi [47]	12	TRIB3, ***SOCS1***, CAV1, SLC7A5, SLC2A3, CDKN2A, G6PD, ASNS, ***AURKA***, ***CISD2***, DDIT4, EGFR
FRG_4_Cli [48]	5	TRIB3, CAV1, ***AURKA***, AKR1C3, SLC7A11
FRG_5_Xfan [49]	17	ASNS, ATG5, ***AURKA***, BAP1, FTH1, BNIP3, ***CISD2***, ***SOCS1***, DRD4, FBXW7, ***LINC00336***, MAP1LC3A, MAP3K5, PRDX6, ***ZFP69B***, SLC7A5, SLC2A3
FRG_6_Wlu [50]	4	TRIB3, ***FTH1***, SLC2A3, BNIP3
FRG_7_HZhu [51]	3	CA9, TNFAIP3, NRAS
FRG_8_Zhuang [52]	7	ATG5, CDKN2A, MAP3K5, OTUB1, SLC2A3, ***SOCS1***, TRIB3
FRG_9_Gshan [53]	15	***NOX1***, CD44, TP63, ***NOX3***, EPAS1, MYB, ***CDO1***, DUOX2, EGFR, SLC7A11, ***AKR1C2***, ***PROM2***, SQSTM1, ***CYBB***, ***FTH1***
FRG_10_Lxu [54]	6	ATG5, PRDX6, OTUB1, ***FTH1***, ***SOCS1***, MAP3K5
FRG_11_Dhe [55]	10	***MAP1LC3A***, SLC7A5, OTUB1, PRDX6, MAP3K5, ***SOCS1***, ATG5, DDIT4, ACSL3, PRKAA2
FRG_12_Qli [56]	6	TRIB3, **SOCS1**, FLT3, IL6, KEAP1, NQO1
FRG_13_Fhan [57]	3	***FTH1***, PHKG2, TFRC

#### 2.2.2. Cox Regression Analyses in the Two Gene Expression Datasets

The 13 selected ferroptosis signatures were imputed using the hacksig (version 0.2.0) R package as implemented in https://github.com/Acare/hacksig (accessed on 11 September 2024) [58] in both gene expression datasets separately. Following the specific algorithm defined for each signature, a signature score was generated. The primary clinical endpoint is overall survival (OS), defined as the time from diagnosis to death. Any patients lost to follow-up or still alive at the time of evaluation were censored. Univariate Cox regression analysis was then performed for each signature to assess the effect of the ferroptosis-associated signatures on OS using the survival R package [59,60] (version 3.5.8), and the results (hazard ratio, 95% confidence interval, and *p*-value) were visualized using forest plots generated with the ggplot2 R package (version 3.5.1). We focused only on signatures with significant *p*-values (less than 0.05) in each dataset for further analyses.

#### 2.2.3. Analysis of Association of Signatures with Risk Clusters

The scores of the selected signatures (previously calculated by hacksig) were analyzed, separating the two gene expression datasets according to the three risk clusters identified in the 286 OPSCC HPV-positive cases [8]. Data were visualized through boxplots using the ggplot2 R package [61] (version 3.5.1). The Wilcoxon test was applied to assess the difference in the distribution of the signature scores between the clusters.

#### 2.2.4. Analysis of Association of Signatures with Tumor Microenvironment

The abundance of immune and stromal cells within the tumor microenvironment was assessed using the xCell R package (version 1.1.0) [62], a tool designed to quantify cell type composition from bulk gene expression data. xCell utilizes single-sample Gene Set Enrichment Analysis (ssGSEA) to estimate the relative abundance of 64 distinct cell types, which encompasses a wide range of immune cells (both adaptive and innate), hematopoietic progenitors, epithelial cells, as well as extracellular matrix (ECM) cells. This methodology is powered by an innovative compendium of 489 gene sets, each representing a specific cell type or biological function, making it a robust approach for studying complex cellular landscapes in various tissue types. For our analysis, we specifically focused on the cell types that correspond to three primary summary scores provided by the xCell tool: (i) the immune score; (ii) the stroma score; and (iii) the microenvironment score, which gives an overall measure of the cellular composition within the tissue. Correlation plots were created using the corrplot R package (https://cran.r-project.org/web/packages/corrplot/vignettes/corrplot-intro.html, accessed on 11 November 2024).

#### 2.2.5. Prediction of the Responsiveness to Target and Chemotherapeutic Treatments

To evaluate how variations in ferroptosis signatures influence sensitivity to both targeted therapies and chemotherapeutic agents, the pRRophetic R package (version 0.5) was employed. This tool facilitated the prediction of the half-maximal inhibitory concentration (IC50) values for a total of 138 distinct drugs, providing insights into their efficacy and potential therapeutic impact [63].

## 3. Results

### 3.1. Ferroptosis Gene Expression Selection

A comprehensive literature survey was conducted to investigate the biological significance of ferroptosis in patients with HPV-positive HNSCC. During this analysis, a total of thirteen ferroptosis-related gene expression signatures, all derived from HNSCC datasets within The Cancer Genome Atlas (TCGA), were identified, and the algorithms retrieved from the original papers. As a result, these ferroptosis signatures were selected for further analysis, and their key characteristics are summarized in Supplementary Table S1.

### 3.2. Selection of Clinical Cohorts

Two distinct cohorts, namely Metanalysis-HPV267 and BD2-HPV286, which included both gene expression data and corresponding clinical annotations, were utilized as testing sets to evaluate the clinical relevance of the 13 selected ferroptosis signatures. In both cohorts, the majority of patients were male, comprising 83% of the “Metanalysis-HPV267” cohort and 76% of the “BD2-HPV286” cohort. However, a statistically significant difference was observed, with a higher prevalence of females in the “BD2-HPV286” cohort (*p* = 0.0289). The median ages for the two cohorts were 58.1 and 60.5 years, respectively.

When analyzing the cohorts according to the TNM staging system, as defined by the 7th edition, the “Metanalysis-HPV267” cohort exhibited 9.77% of patients classified as stage I, a stage not represented in the “BD2-HPV286” cohort. In contrast, when the more recent TNM 8th edition was applied, 46% of patients in both cohorts were classified as stage I. For stage II, 24% and 27% of patients from the “Metanalysis-HPV267” and “BD2-HPV286” cohorts, respectively, were identified, while 21% and 27% of patients were categorized as stage III. Notably, stage IV cases were present only in the “Metanalysis-HPV267” cohort, which aligns with the inclusion criteria of the BD2-DECIDE project, which selected patients treated with a curative intent. No significant differences were found between the cohorts regarding smoking status or follow-up period.

A detailed summary of the clinical characteristics for both cohorts is provided in Table 2.

### 3.3. Prognostic Role of the 13 Selected Ferroptosis Signatures

**Figure 2:**
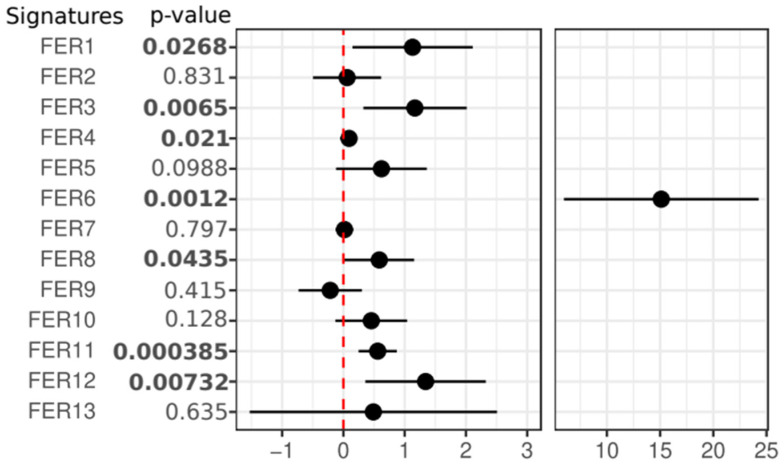
Forest plot of Cox regression analysis for the 13 selected ferroptosis signatures in the Metanalysis-HPV267 dataset. Patients were associated with OS as a clinical endpoint with each signature as a continuous trait. A significant HR > 0 was found for FER1, FER3, FER4, FER6, FER8, FER11, and FER12. Data are reported as log2(HR) and 95% confidence intervals.

**Figure 3:**
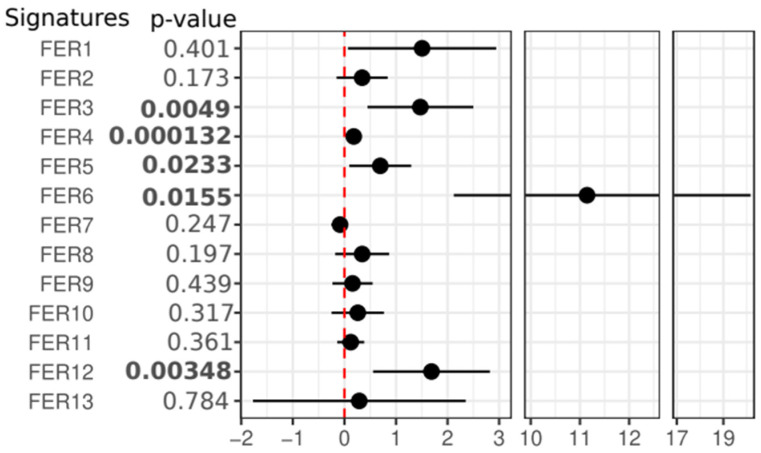
Forest plot of Cox regression analysis for the 13 selected ferroptosis signatures in the BD2-HPV286 dataset. A significant HR > 0 was found for FER3, FER4, FER5, FER6, and FER12. Patients were associated with OS as a clinical endpoint with each signature as a continuous trait. Data are reported as log2(HR) and 95% confidence intervals.

Using overall survival (OS) as the clinical endpoint, the 13 selected ferroptosis signatures were evaluated for their association with clinical outcomes in the Metanalysis-HPV267 and BD2-HPV286 cohorts. In the Metanalysis-HPV267 dataset, seven signatures (FER1, FER3, FER4, FER6, FER8, FER11, and FER12) were found to be significantly associated with OS (*p* ≤ 0.05), with all showing a positive correlation, indicated by a log(HR) greater than 0 (Figure 2 and Supplementary Table S3). In the BD2-HPV286 dataset, four out of the seven signatures that were significant in Metanalysis-HPV267 (FER3, FER4, FER6, and FER12) were consistently confirmed to be significantly associated with OS, each having *p*-values less than 0.05 and log(HR) greater than 0. However, three of the signatures (FER1, FER8, and FER11) failed to replicate their significant association with OS in the BD2-HPV286 cohort. Additionally, FER5 was found to show a significant association with OS, but only in the BD2-HPV286 dataset (Figure 3 and Supplementary Table S4). These results highlight both the robustness and variability of some of the ferroptosis signatures across different patient cohorts, while others failed.

### 3.4. Association of Ferroptosis Signatures with HPV-Pos OPSCC Risk Clusters

**Figure 4:**
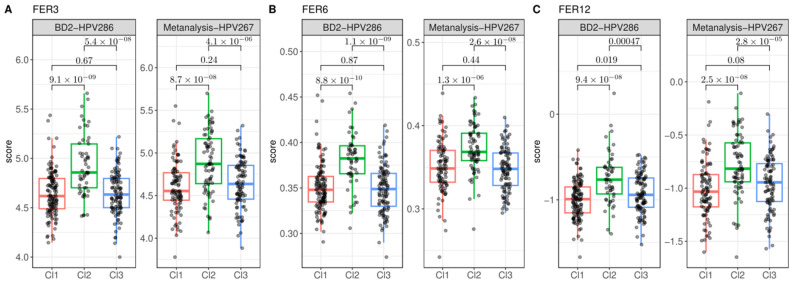
Comparison of the ferroptosis signatures with significant scores on the high-risk cluster (Cl2) in both the Metanalysis-HPV267 and BD2-HPV286 datasets. The tested signatures show significantly higher scores associated with Cl2 (high risk) compared to Cl1 (low risk) and Cl3 (intermediate risk). (**A**) Correlation of FER3 and prognostic clusters in BD2-HPV286 and Metanalysis-HPV267. (**B**) Correlation of FER6 and prognostic clusters in BD2-HPV286 and Metanalysis-HPV267. (**C**) Correlation of FER12 and prognostic clusters in BD2-HPV286 and Metanalysis-HPV267.

**Figure 5:**
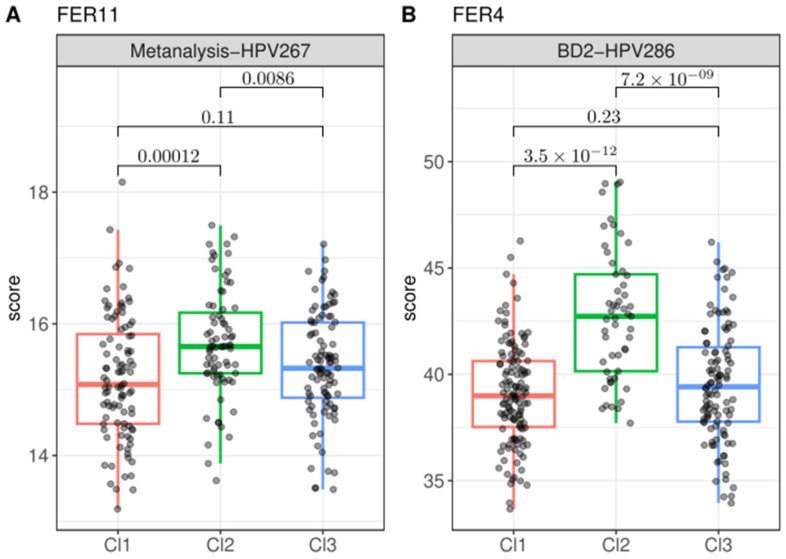
Ferroptosis signatures with significant scores on the high-risk cluster (Cl2) only in one dataset. The tested signatures show significantly higher scores associated with Cl2 (high risk) compared to Cl1 (low risk) and Cl3 (intermediate risk) (**A**) FER11 in the Metanalysis-HPV267 dataset. (**B**) FER4 in the BD2-HPV286 dataset.

The ferroptosis gene expression signatures were analyzed in relation to the three prognostic clusters (Cl1, Cl2, and Cl3) previously defined by our group [8]. Among these clusters, Cl2, which is associated with the worst prognosis in the BD2-HPV286 cohort, exhibited significantly higher scores for the signatures FER3, FER6, and FER12 compared to both Cl1 and Cl3 in both datasets (Figure 4A–C). These findings suggest that the expression of these ferroptosis signatures is more strongly associated with poor prognosis in Cl2. Furthermore, for signatures FER4 and FER11, significantly higher scores were observed in Cl2 compared to the other clusters, but this trend was only consistent in one of the datasets for each signature: FER11 was significantly upmodulated in Cl2 in the Metanalysis-HPV267 dataset, while FER4 showed significantly higher scores in Cl2 in the BD2-HPV286 dataset (Figure 5). In contrast, no significant differences in ferroptosis signature scores were observed between Cl1 and Cl3 in either cohort, indicating that these clusters share similar gene expression profiles with respect to ferroptosis-related genes. These results suggest a potential link between specific ferroptosis signatures and the most aggressive tumor phenotypes, particularly within Cl2, which may help refine prognostic models for HNSCC patients.

**Figure 6:**
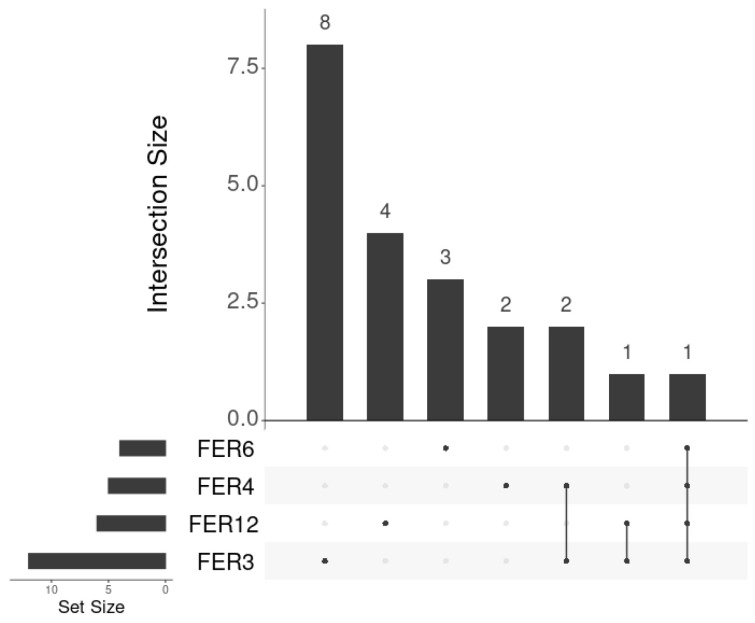
Overlap of the genes in the selected signatures. The bars on the bottom left represent the number of genes in each signature. The bars on the plot represent the number of genes in common between the signatures marked with black points on the panel below.

Moreover, we considered how many genes were in common among the significant ferroptosis signatures to understand if these genes influenced the signature’s trend. Figure 6 illustrates the overlap of genes among the selected signatures. Three genes (i.e., AURKA, CAV1, and TRIB3) are shared between FER3 and FER4; two genes (i.e., SOCS1 and TRIB3) are in common between FER3 and FER12, while only one gene (i.e., TRIB3) is in common to all four signatures. Since AURKA and SOCS1 were filtered out, as explained in Section 2.2.1, only TRIB3 and CAV1 were identified as genes with the most impact among the signatures and investigated in deep detail.

### 3.5. Characteristics of Genes Present in Signatures

Taking into consideration the ferroptosis signatures that were significant in both datasets, as well as their association with prognostic clusters, we assessed the role of the genes commonly expressed across these datasets and present in the selected signatures. TRIB3 was identified as a common gene in the signatures FER3, FER4, FER6, and FER12, while CAV1 was shared between FER3 and FER4. Although the role of TRIB3 had not been fully elucidated in the existing ferroptosis gene database, there is evidence in the literature suggesting its role as a suppressor of ferroptosis [64,65]. On the other hand, CAV1 was already recognized as a suppressor in the ferroptosis gene database (Table 3).

**Figure 7:**
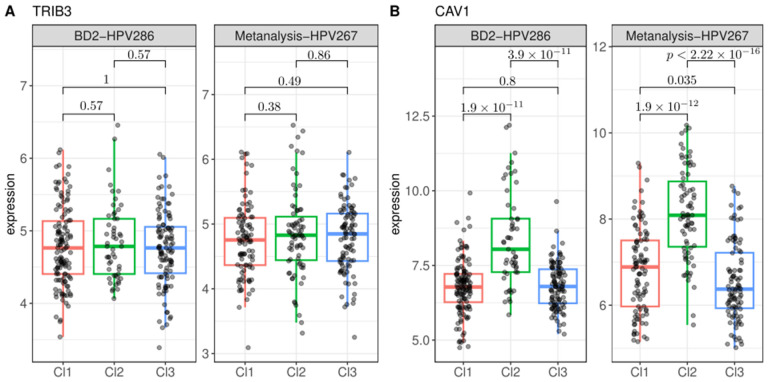
Expression levels of TRIB3 (**A**) and CAV1 (**B**) associated with prognostic clusters in the Metanalysis-HPV267 and BD2-HPV286. No significant association was found for TRIB3, while CAV1 is upregulated in Cl2 compared to Cl1 and Cl3.

We then measured the expression levels of these genes across the three prognostic clusters in both the Metanalysis-HPV267 and BD2-HPV286 datasets. The resulting box plots revealed that TRIB3 was expressed in both datasets, but its expression levels did not significantly differ across the three clusters (Figure 7A). In contrast, CAV1 was expressed in all three clusters in both datasets, with its expression being notably higher in the worst-prognosis cluster, Cl2, in both datasets (Figure 7B).

### 3.6. Correlation of Ferroptosis Signature and Immune Profile

A comprehensive correlation analysis was conducted to examine the relationship between the ferroptosis signatures and the immune microenvironment, specifically assessing whether ferroptosis is associated with the cancer immune profile. This analysis was performed using the xCell scores, which quantify various immune and stromal components within the tumor microenvironment.

**Figure 8:**
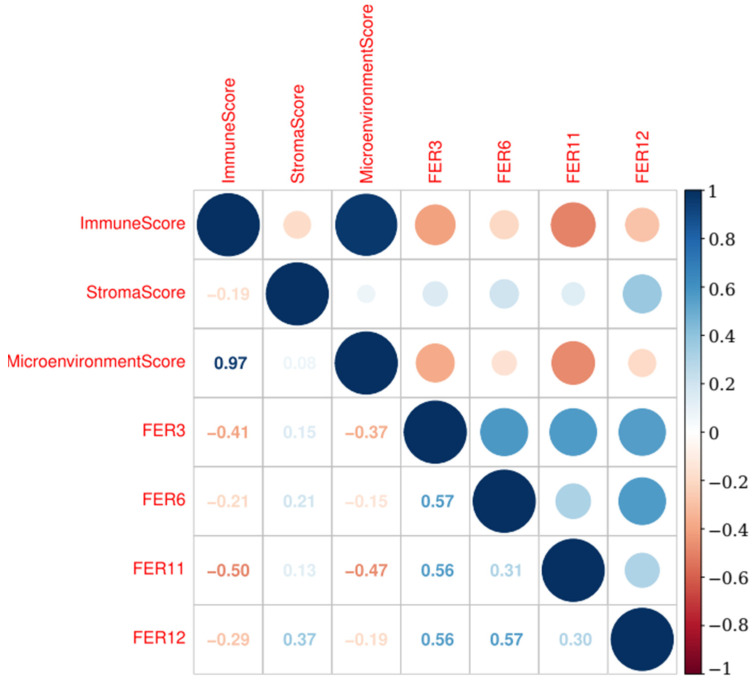
Correlation plot between the selected ferroptosis signatures and the immune, microenvironment, and stroma scores in the Metanalysis-HPV267 cohort. A negative correlation was found between immune score and ferroptosis signatures. The plot depicts the level of correlation by dot size. The bar shows the correlation ranging from 1 to −1 as a color scale.

In the Metanalysis-HPV267 cohort, we observed that the ferroptosis signatures FER3, FER6, FER11, and FER12 exhibited negative correlations with both the immune score and the microenvironment score. Notably, FER3 and FER11 demonstrated the strongest inverse associations, indicating that higher expression levels of these ferroptosis-related genes were associated with lower immune and microenvironmental activity. In contrast, these same signatures, particularly FER3, FER6, FER11, and FER12, showed a slight positive correlation with the stroma score, suggesting a potential association with stromal components in the tumor microenvironment. Furthermore, all ferroptosis signatures were positively correlated with each other, with particularly strong positive correlations observed between FER3 and FER6, as well as FER3 and FER11. Similarly, FER6 and FER12 also displayed a robust positive correlation (Figure 8).

**Figure 9:**
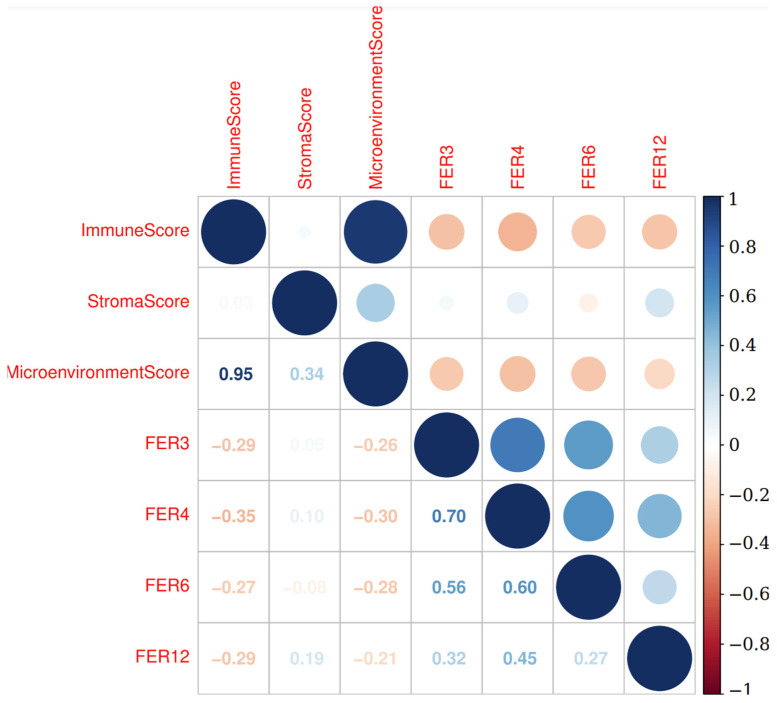
Correlation plot between the selected ferroptosis signatures and the immune, microenvironment, and stroma scores in the BD2-HPV286 cohort. A negative correlation was found between immune score and ferroptosis signatures. The plot depicts the level of correlation by dot size. The bar shows the correlation ranging from 1 to −1 as a color scale.

In the BD2-HPV286 cohort, the results mirrored those observed in the Metanalysis-HPV267 dataset. Specifically, FER3, FER4, FER6, and FER12 were negatively correlated with both the immune and microenvironment scores, further reinforcing the association between ferroptosis and a diminished immune and microenvironmental profile. Additionally, these signatures were positively correlated with the Stroma score, aligning with the findings from the Metanalysis-HPV267. Among the ferroptosis signatures, FER3, FER4, FER6, and FER12 demonstrated strong positive intercorrelations, with the most notable positive associations occurring between FER3, FER4, and FER6, as well as between FER4 and FER12 (Figure 9).

To further investigate the relationship between ferroptosis signatures and components of the immune microenvironment, we calculated the correlations among these variables, as shown in Supplementary Figure S1. Most immune populations (B-cells, CD4+ T-cells, CD8+ T-cells, dendritic cells, eosinophils, MPP, macrophages, and NK cells) exhibited a negative correlation in both the Metanalysis-HPC267 and BD2-HPV286 datasets, consistent with the overall negative correlation between ferroptosis signatures and ImmuneScore. Among these populations, B-cells, CD4+ T-cells, and CD8+ T-cells demonstrated stronger negative correlations in the Metanalysis-HPC267 dataset compared to BD2-HPV286. In the latter dataset, some of these correlations were not statistically significant (*p*-value > 0.05). The only components showing a positive correlation were monocytes in BD2-HPV286 and HSC, monocytes, and neutrophils in the Metanalysis-HPC267 dataset.

These findings suggest that certain ferroptosis-related signatures, particularly FER3, FER6, FER11, and FER12, may be linked to a suppressive immune environment while also correlating with stromal components in the tumor microenvironment. This highlights the potential role of ferroptosis in modulating the tumor’s immune landscape and its interaction with the stromal environment, offering insights into the complex relationship between cellular death mechanisms and tumor progression.

### 3.7. Drug Sensitivity Linked to Ferroptosis Signatures

**Figure 10:**
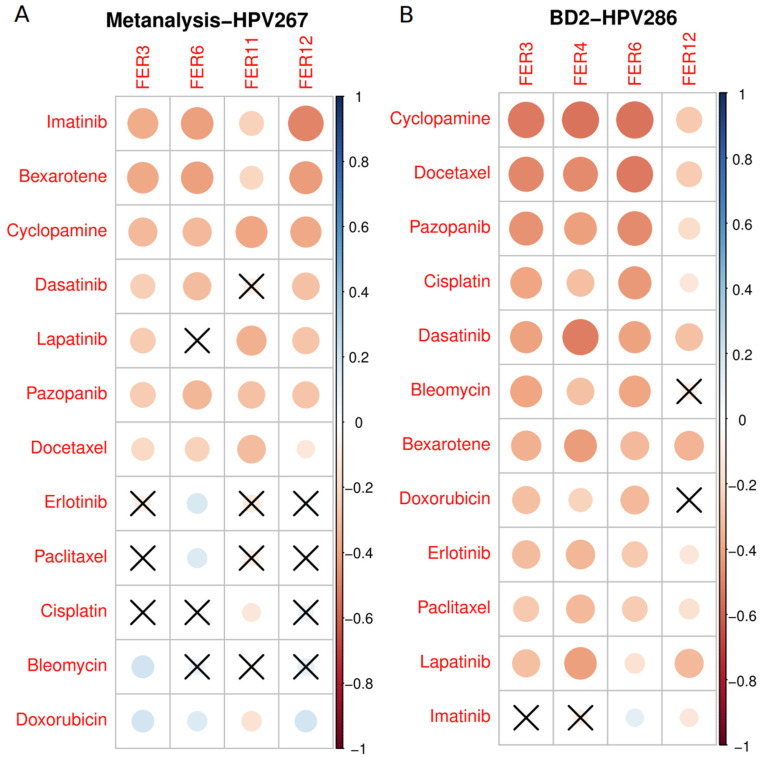
Predicted sensitivity correlation with ferroptosis signatures. IC50 correlations were predicted in the Metanalysis-HPV267 (**A**) and BD2-HPV286 (**B**) datasets. The analysis focused exclusively on targeted and chemotherapeutic compounds approved for clinical use in oncology, excluding experimental drugs. Twelve compounds that exhibited significant negative correlations in both datasets, based on at least one of the selected ferroptosis signatures (i.e., FER3, FER6, FER11, and FER12 for Metanalysis-HPV267; FER3, FER4, FER6, and FER12 for BD2-HPV286), were identified and visualized. The correlation values are reported in Tables S5 and S6 for Matanalysis-HPV267 and BD2-HPV286, respectively. The size of the circles represents the *p*-value, while the color indicates the correlation level. Not significant associations (*p*-value > 0.05) are referred to as X.

To evaluate the potential of ferroptosis in predicting treatment outcomes, we compared the half-maximal inhibitory concentration (IC50) with selected ferroptosis signatures. The IC50 serves as a quantitative measure of a drug’s therapeutic ability to induce ferroptosis in cancer cells, where lower IC50 values indicate higher drug sensitivity. We assessed the therapeutic efficacy of 138 anti-tumor drugs on both the Metanalysis-HPV267 and BD2-HPV286 datasets, excluding drugs with *p* > 0.05 across all selected signatures. As illustrated in Figure 10, the IC50 values of Cyclopamine, Bexarotene, Dasatinib, Pazopanib, and Docetaxel demonstrated a significant inverse correlation with the ferroptosis signatures in the Metanalysis-HPV267 (Figure 10A) and BD2-HPV286 (Figure 10B). These findings suggest that these targeted and chemotherapeutic agents may be particularly effective in patients with poor prognoses linked to ferroptosis-related markers.

## 4. Discussion

The existing literature highlights the critical need for biomarkers that can more accurately define the prognosis and treatment strategies for patients with HPV-positive OPSCC. In response to this need, we aimed to enhance patient stratification by evaluating the prognostic and predictive potential of ferroptosis gene expression models and signatures. Our approach involved assessing ferroptosis-related gene expression signatures that were developed and tested using data from the TCGA HNSCC cohort. We sought to validate these signatures’ performance in two independent cohorts: a Metanalysis of public databases and the BD2 cohort. The selection of these cohorts was driven by several key criteria: (1) inclusion of only HPV-positive HNSCC patients (Metanalysis) or only HPV-positive OPSCC patients (BD2); (2) the inclusion of a sufficiently large sample size, with more than 250 cases in both cohorts; and (3) the homogeneity of clinical parameters, as these were centrally collected within a dedicated dataset established as part of an EU project (BD2 cohort) [41]. The Metanalysis-HPV267 provides the advantage of integrating diverse real-world datasets, offering a broad and comprehensive perspective on ferroptosis-related signatures. Conversely, the BD2-HPV286 clinical cohort enables the validation of findings under controlled experimental conditions, reducing variability and enhancing interpretability. This rigorous cohort selection ensured that the evaluation of ferroptosis signatures was robust, reproducible, and applicable to HPV-positive OPSCC patients specifically, providing valuable insights into the role of ferroptosis in cancer prognosis and treatment.

These parameters enabled the identification of the prognostic role of three ferroptosis signatures (FER3, FER6, and FER12) that resulted in significant results and presented in both the Metanalysis-HPV267 and BD2-HPV286 datasets the same trend and a log(HR) > 0 suggesting an association with poor prognosis in agreement. According to the obtained results, when we correlated these signatures with the already tested and validated prognostic clusters in HPV-pos OPSCCs [8] by our group, we demonstrated that they were associated with the high-risk Cl2. This cluster was characterized in BD2-HPV286 by a low immune score and a high stroma score. When we evaluated the correlation among ferroptosis signatures and the tumor microenvironment, we demonstrated that FER3, FER6, and FER12 were negatively correlated to the immune score in both cohorts.

When, in the original paper [8], the high-risk Cl2 was compared with the other two clusters, it was enriched in epithelial–mesenchymal transition (development), glycolysis (metabolism), UV response (DNA damage), NOTCH signaling, TGF beta signaling, hypoxia (pathway), mitotic spindle, p53 pathway, and MYC target V1 (proliferation). Interestingly, Cl1 and Cl3 exhibited the same level of association to the ferroptosis signatures. This supports the hypothesis that Cl2 is a distinct subgroup, while Cl1 and Cl3 represent two ‘shades’ of a relatively favorable disease: Cl1 being highly favorable and Cl3 moderately favorable.

These results confirmed that FER3, FER6, and FER12 were associated with poor prognosis and impaired tumor microenvironments, suggesting that ferroptosis could have a strict relationship with the tumor microenvironment. Therefore, in the future it should be important to study how ferroptosis can influence it and if their perturbation could affect the tumor progression.

Considering the same role of FER3, FER6, and FER12 in both cohorts, we evaluated the expression of genes in common among these significant signatures. TRIB3 was shared among FER3, FER6, and FER12. There is recent evidence in the literature that demonstrates the ferroptosis suppression role of TRIB3. TRIB3 is a stressor sensor involved in the alterations of the cellular microenvironment and various stress-inducing factors, including insulin, glucose deficiency, and the accumulation of misfolded proteins within the endoplasmic reticulum [66], and it has a tumorigenic role in different cancers [67,68,69,70,71].

In addition, CAV1 was considered because it is common in FER3 and FER4, the most significant signatures in BD2-HPV286. CAV1 was defined by the ferroptosis genes database as a suppressor, and TRIB3 was more expressed in Cl2 than in the low- and intermediate-risk clusters. CAV1 is an integral membrane protein involved in cell signaling and transport [72,73] and regulates cell proliferation, invasion, cell death, and lipid metabolism. It has been demonstrated that its overexpression was associated with an aggressive phenotype leading to poor prognosis in HNSCC patients [74]. In addition, high expression of CAV1 predicted worse prognosis in OSCC, can inhibit ferroptosis and cisplatin sensitivity in cancer cells, promoting migration and invasion, and can affect the immune cell infiltration [75]. The common expression of TRIB3 in FER3, FER6, and FER12 and CAV1 in FER3 and FER4, which are all significant and majorly expressed in the cluster with the worst prognosis, Cl2, suggests that these two genes have an important biological impact, considering their ferroptosis suppressor role. However, we have confirmed this hypothesis only for CAV1 because it resulted in high expression in the Cl2.

In the context of new treatments, it has been demonstrated that the silencing of TRIB3 using the molecular inhibitor hesperidin in HNSC induced ferroptosis and promoted cell death, suggesting that this gene could be a target for an innovative therapeutic strategy for HNSCC. Moreover, TRIB3, which is highly expressed in prostate cancer, can be targeted by Palbociclib, a CDK4/6 inhibitor approved for the treatment of breast cancer, augmenting the response of prostate cancer cells to ferroptosis inducers [76]. Moreover, CAV1 is a ferroptosis-related antigen and was positively correlated with dendritic cells and macrophage expression and negatively correlated with B-cell expression; therefore, it could be a potential target for patients with an immune cold profile [77]. Therefore, in view of these results, the weight of TRIB3 and CAV1 in the tested and significant signatures will be evaluated in order to give other evidence of their biological role in ferroptosis.

To evaluate the potential of ferroptosis signatures in predicting treatment outcomes, we analyzed the half-maximal inhibitory concentration (IC50) values of various anti-tumor agents in relation to selected ferroptosis signatures. Our analysis included 138 anti-tumor drugs tested on the Metanalysis-HPV267 and BD2-HPV286 datasets. We specifically focused on clinically approved targeted and chemotherapeutic compounds. Drugs with *p*-values > 0.05 across all selected ferroptosis signatures were excluded to ensure robustness. Among the remaining compounds, cyclopamine, bexarotene, dasatinib, pazopanib, and docetaxel showed significant inverse correlations with ferroptosis signatures, highlighting their therapeutic relevance. Cyclopamine, a Hedgehog pathway inhibitor, has demonstrated potential anti-tumor activity, particularly in malignancies with aberrant Hedgehog signaling [78]. Its correlation with ferroptosis signatures suggests an additional mechanism of action that could be exploited in treatment strategies. Bexarotene, a retinoid X receptor (RXR) agonist, is known for its use in cutaneous T-cell lymphoma [79]. Its association with ferroptosis sensitivity indicates a possible application in broader cancer types characterized by ferroptosis-related vulnerabilities. Dasatinib, a multi-target tyrosine kinase inhibitor, is primarily employed in chronic myeloid leukemia and other malignancies driven by BCR-ABL1 or SRC family kinases [80]. Its inverse correlation with ferroptosis signatures supports its potential utility in ferroptosis-prone tumors. Pazopanib, a VEGFR and PDGFR inhibitor, is approved for renal cell carcinoma and soft tissue sarcoma [81]. The observed sensitivity in ferroptosis-related contexts aligns with its capacity to disrupt tumor angiogenesis and metabolic stress responses. Docetaxel, a well-established chemotherapeutic agent targeting microtubules, has broad applications in solid tumors, including breast, prostate, and lung cancers [82]. Its significant inverse IC50 correlation with ferroptosis signatures suggests enhanced efficacy in ferroptosis-sensitive malignancies. These findings emphasize the therapeutic potential of targeting ferroptosis-related pathways using both targeted agents and chemotherapeutics. Future studies should explore the mechanisms underlying these correlations to refine treatment strategies and optimize patient outcomes.

The study was not exempt from limitations. In particular, the Metanalysis-HPV267 includes some HPV-positive cases without the information about the subsite. We have noticed that the correlation among signatures in the Metanalysis-HPV267 was higher than in BD2-HPV286. Consequently, the variation in primary site across the databases analyzed suggests that the location of the primary site may have influenced the results. In addition, the gene expression of both datasets was performed using an array method, and for this reason we lost the expression of some genes. In fact, among the common genes between the signatures FER3 and FER4, there was AURKA, which was filtered out during data processing, likely due to its low expression levels, but different authors discovered that it had a suppressor role [83,84]. This gene could also be important to study, but, for the reason explained above, its expression was not detected in our gene expression matrices. Despite these limits, one of the strengths of our study was the sample size and the accuracy in the case selection. Moreover, net of the limitations cited above, these results may have a relevant impact for clinical management of HPV-positive OPSCC patients. Signatures with prognostic significance could suggest a treatment intensification in cases predicted to have unfavorable outcomes. The in silico prediction of drug sensitivity of the analyzed transcriptomic models may guide the design of future precision oncology trials with drugs acting on deregulated ferroptotic pathways.

## 5. Conclusions

Taking all the results into account, we validated ferroptosis signatures in large HPV-positive OPSCC datasets, and we assessed the importance of ferroptosis suppressor genes in the progression of the disease. Our study represents the first comprehensive evaluation of ferroptosis in HPV-positive OPSCC. Future research is warranted to further investigate and validate our findings. Ferroptosis-related signatures should be examined in additional HPV-positive OPSCC datasets, following the workflow established in this study. Such efforts will help confirm the biological significance of these signatures, their association with the tumor microenvironment, and, ultimately, facilitate the translation of this knowledge into clinical practice. Ferroptosis could gain the stratification of HPV-positive OPSCC patients, and in the future, it could be targeted to improve patient outcomes.

## Figures and Tables

**Table 2:** Analyzed cohorts of HPV-HNSCC patients. Clinical characteristics of patients with informative transcriptomics.

	Metanalysis-HPV267 (%)	BD2-HPV286 (%)	*p*-Value (Fisher Test)
Nr of patients	267	286	
Sex at birth (Men/Women) Not available	221 (83)/45 (16) 1	216 (76)/70 (24) -	0.0316
Median age at diagnosis (range) Not available	58.1 years (35–80) 10	60.5 years (40–86) -	
Stage (TNM 7th) I–II III IVa IVb Not available	24 (9) 49 (18) 175 (66) 16 (6) 3 (1%)	- 49 (17) 205 (72) 32 (11) -	2.061 × 10^−8^
Stage (TNM 8th) I II III IVa/IVb Not available	123 (46) 65 (24) 55 (21) 19 (7) 5 (2%)	132 (46) 77 (27) 77 (27) - -	8.162 × 10^−7^
Smoking Current or former Never Unknown	165 (62) 84 (31) 18 (7)	192 (67) 87 (30) 7 (2)	0.0425
Tumor subsite Oropharynx Oral cavity Larynx–Hypopharynx Not available	190 (71) 16 (6) 11 (4) 50 (19)	286 (100) - - -	5.895 × 10^−28^
Median follow-up (range)	36.1 (1–224.4)	43.3 (1.64–121.2)	

**Table 3:** Gene list of ferroptosis signatures and role classification in driver or suppression.

Signature ID	#Genes	Genes	Drivers	Suppressors	Unclassified	Marker	Not Found
FRG_1_FHe	7	*KEAP1*, *CDKN2A*, *EIF2S1*, *FTH1*, *MAP3K5*, *SELENOS*, *SKC2A3*	*KEAP1* (4), *CDKN2A* (ARF)	*FTH1* (4)	*SELENOS*, *MAP3K5*, *EIF2S1*	FTH1 (1)	SKC2A3
FRG_2_	10	ATG5, *BID*, *ACO1*, *GOT1*, *AKR1C3*, *GLS2*, *AKR1C3*, *ALOX15*, *SCO2*, *MAP1LC3A*, *MAP3K5*	*ATG5* (2),	*AKR1C3*,	*MAP1LC3A*, *MAP3K5*		
FRG_3_SLi	12	*TRIB3*, *SOCS1*, *CAV1*, *SLC7A5*, *SLC2A3*, *CDKN2A*, *G6PD*, *ASNS*, *AURKA*, *CISD2*, *DDIT4*, *EGFR*	*CDKN2A* (ARF), *SOCS1*, *G6PD*, *EGFR*	*CAV1*, *G6PD*, *CISD2*	*AURKA*, *SLC7A5*, *ASNS*, *SLC2A3*, *DDIT4*, *TRIB3*		
FRG_4_Cli	5	*TRIB3*, *CAV1*, *AURKA*, *AKR1C3*, *SLC7A11*	*SLC7A11*	AKR1C3, CAV1	*AURKA*, *TRIB3*		
FRG_5_Xfan	17	*ASNS*, *ATG5*, *AURKA*, *BAP1*, *FTH1*, *BNIP3*, *CISD2*, *SOCS1*, *DRD4*, *FBXW7*, *LINC00336*, *MAP1LC3A*, *MAP3K5*, *PRDX6*, *ZFP69B*, *SLC7A5*, *SLC2A3*	*ATG5* (2) *SOCS1*, *BAP1*, *FBXW7*	*FTH1* (4), *CISD2*, *LINC00336*, *PRDX6*	*AURKA*, *BNIP3*, *DRD4*, *ZFP69B*, *SLC7A5*, *SLC2A3*, *MAP3K5*, *MAP1LC3A*, *ASNS*	FTH1 (1)	
FRG_6_Wlu	4	*TRIB3*, *FTH1*, *SLC2A3*, *BNIP3*		*FTH1* (4)	*BNIP3*, *SLC2A3*, *TRIB3*	FTH1 (1)	
FRG_7_	3	*CA9*, *TNFAIP3*, *NRAS*					
FRG_8_Zhuang	7	*ATG5*, *CDKN2A*, *MAP3K5*, *OTUB1*, *SLC2A3*, *SOCS1*, *TRIB3*	*ATG5* (2), *CDKN2A* (ARF), *SOCS1*, *OTUB1*		*SLC2A3*, *TRIB3*, *MAP3K5*		
FRG_9_Gshan	15	*NOX1*, *CD44*, *TP63*, *NOX3*, *EPAS1*, *MYB*, *CDO1*, *DUOX2*, *EGFR*, *SLC7A11*, *AKR1C2*, *PROM2*, *SQSTM1*, *CYBB*, *FTH1*		*FTH1* (4),			
FRG_10_Lxu	6	*ATG5*, *PRDX6*, *OTUB1*, *FTH1*, *SOCS1*, *MAP3K5*	*ATG5* (2), *OTUB1* (2), *SOCS1*	*PRDX6*,	*MAP3K5*	FTH1 (1)	
FRG_11_Dhe	10	*MAP1LC3A*, *SLC7A5*, *OTUB1*, *PRDX6*, *MAP3K5*, *SOCS1*, *ATG5*, *DDIT4*, *ACSL3*, *PRKAA2*	*SOCS1*, *ATG5*, *PRKAA2* (2)	*OTUB1*, *PRDX6*, *ACSL3*, *PRKAA2* (1)	*SLC7A5*, *DDIT4*, *MAP3K5*, *MAP1LC3A*		
FRG_12_Qli	6	*TRIB3*, *SOCS1*, *FLT3*, *IL6*, *KEAP1*, *NQO1*	*KEAP1* (4), *SOCS1*, *IL6* (2), *FLT3*	*NQO1*, *IL6* (1)	*TRIB3*		
FRG_13_	3	*FTH1*, *PHKG2*, *TFRC*		*FTH1* (4)			

## Data Availability

The IDs and links to the public repositories for the original data have been provided in the Supplementary section. The gene expression data for our proprietary cohort was deposited on Gene Expression Omnibus (GEO) under the following ID: GSE262372.

## Supplementary Materials

The following supporting information can be downloaded at https://www.mdpi.com/article/10.3390/cancers17030530/s1, Table S1: Ferroptosis signatures; Table S2: Datasets for Metanalysis-HPC267; Table S3: Cox regression analysis for 13 selected ferroptosis signatures in the Metanalysis-HPV267 dataset; Table S4: Cox regression analysis for 13 selected ferroptosis signatures in the BD2-HPV286 dataset; Table S5: Correlation values between ferroptosis signatures and drugs in the Metanalysis-HPV267 dataset; Table S6: Correlation values between ferroptosis signatures and drugs in the BD2-HPV286 dataset; Figure S1: Correlation between ferroptosis signatures and components of the immune microenvironment.
