# Supplementary material for: Ferroptosis-Related Gene Signatures: Prognostic Role in HPV-Positive Oropharyngeal Squamous Cell Carcinoma
Source: Cancers (Basel). 2025 Feb 5;17(3):530. doi: 10.3390/cancers17030530 (PMC11817470; doi:10.3390/cancers17030530)
Supplement: Supplementary file 1 [file cancers-17-00530-s001.zip › Supplementary Figure S1.pdf]

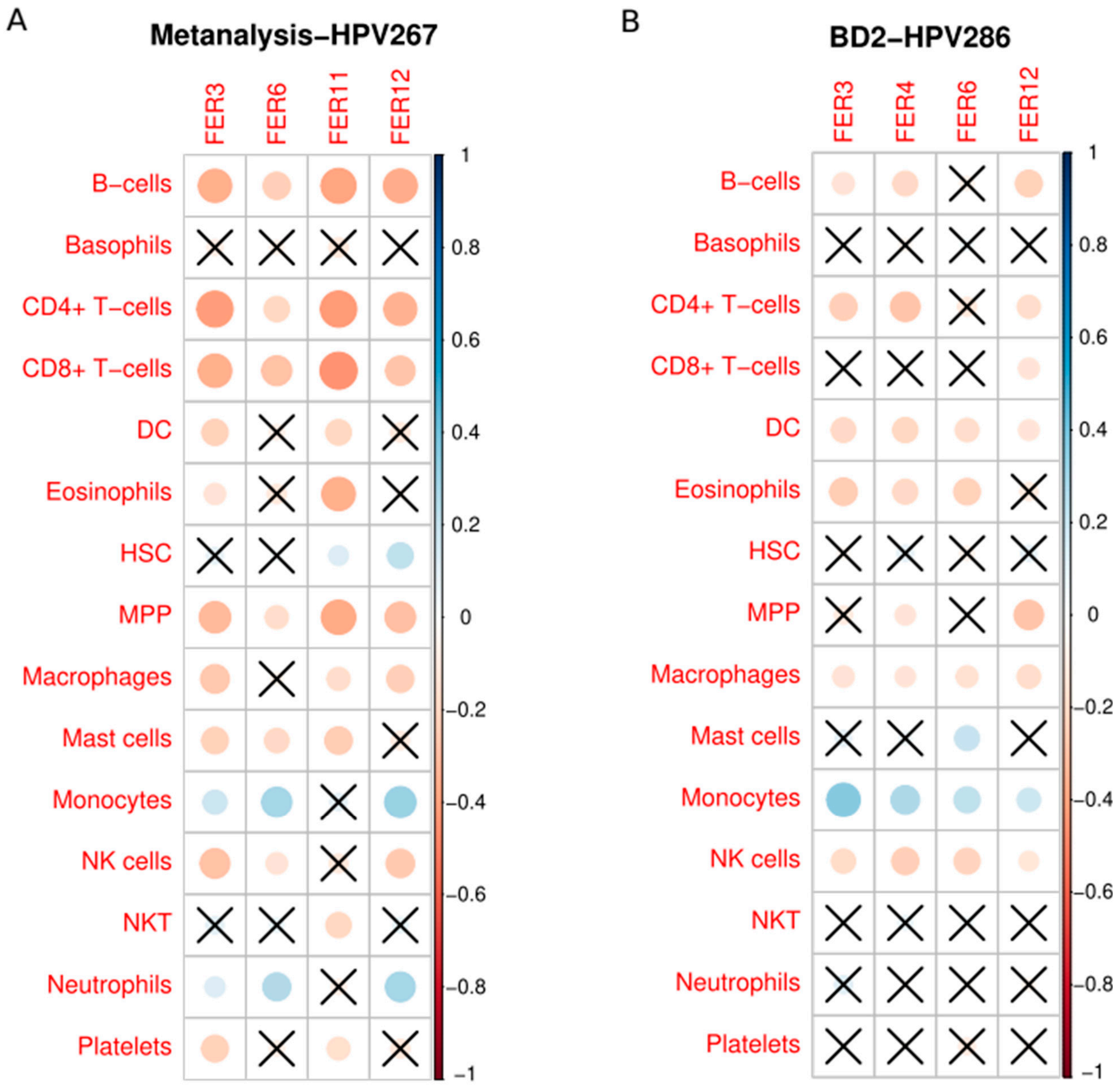


**Supplementary Figure S1.** Correlation between ferroptosis signatures and components of the immune microenvironment. The size of the circles represents the p-value, while the color indicates the correlation level. X corresponds to not significant correlation (p-value > 0.05).
