# Supplementary material for: Ferroptosis-Related Gene Signatures: Prognostic Role in HPV-Positive Oropharyngeal Squamous Cell Carcinoma
Source: Cancers (Basel). 2025 Feb 5;17(3):530. doi: 10.3390/cancers17030530 (PMC11817470; doi:10.3390/cancers17030530)
Supplement: Supplementary file 1 [file cancers-17-00530-s001.zip › TableS1.pdf]

Table S1: Ferroptosis signatures

Signature ID	Signature num	Signature ID in Hacksig package	Typer of tumor	Description	Reference	doi	#genes
FRG_1_FHe	FER1	he2021_ferroptosis_a	HNSCC	*differentially expressed FRGs in TCGA-HNSCC vs. adjacent non-cancerous tissues* ***immune TME analysis***	Feinan He (2021)	10.1016/j.intimp.2021.107789	**7**
FRG_2_HLi	FER2	li2021_ferroptosis_a	Oral cavity squamous cell carcinoma	*differentially expressed FRGs in TCGA-OCSCC vs. adjacent non-cancerous tissues*	Hongyu Li (2021)	10.1186/s12885-021-08478-0	**10**
FRG_3_SLi	FER3	li2021_ferroptosis_b	HNSCC	*differentially expressed FRGs in TCGA-HNSCC vs. adjacent non-cancerous tissues*	Sijia Li et al. (2021)	10.21203/rs.3.rs-349666/v2	**12**
FRG_4_CLi	FER4	li2021_ferroptosis_c	HNSCC	*differentially expressed FRGs in TCGA-HNSCC vs. adjacent non-cancerous tissues* ***immune TME analysis***	Chunyan Li. (2021)	10.3389/fgene.2021.698040	**5**
FRG_5_XFan	FER5	fan2021_ferroptosis	HNSCC	prognostic-related differentially expressed FRGs in TCGA-HNSCC **immune TME analysis**	Xin Fan (2021)	10.3389/fgene.2021.732211	**17**
FRG_6_WLu	FER6	lu2021_ferroptosis	HNSCC	*differentially expressed FRGs in TCGA-HNSCC vs. adjacent non-cancerous tissues* ***immune TME analysis drug sensitivity analysis***	Wei Lu. (2021)	10.3389/fgene.2021.755486	**4**
FRG_7_HZhu	FER7	zhu2021_ferroptosis	Oral cavity squamous cell carcinoma	*differentially expressed FRGs in four OCSCC datasets vs. adjacent non-cancerous tissues*	Haisheng Zhu (2021)	10.1002/2211-5463.13348	**3**
FRG_8_ZHuang	FER8	huang2022_ferroptosis	HNSCC	*differentially expressed FRGs in TCGA-HNSCC*	Zongwei Huang et al. (2022)	10.1016/j.intimp.2021.108431	**7**

				vs. adjacent non-cancerous tissues ***immune TME analysis***			
FRG_9_GShan	FER9	shan2021_ferroptosis	Pancancer	multi-omic and drug sensitivity data of cancer cell lines associated with ferroptosis ,construction of a model applied to **pancancer**	Guangyao Shan et al. (2021)	10.3389/fcell.2021.794475	**12**
FRG_10_LXu	FER10	xu2021_ferroptosis	HNSCC	Identification of Prognostic FRGs in TCGA-HNSCC **immune TME analysis**	Li Xu et al. (2021)	10.1155/2021/5759927	**6**
FRG_11_DHe	FER11	he2021_ferroptosis_b	HNSCC	*differentially expressed FRGs in TCGA-HNSCC* ***immune TME analysis***	Dongsheng He et al. (2021)	10.3389/fcell.2021.739011	**10**
FRG_12_QLi	FER12	li2021_ferroptosis_d	HNSCC	*differentially expressed FRGs in TCGA-HNSCC* ***immune TME analysis***	Qun Li et al. (2021)	10.2147/IJGM.S343233	**6**
FRG_13_FHan	FER13	han2021_ferroptosis	Larynx squamous cell carcinoma	***differentially expressed selected FRGs in TCGA-LCSCC vs. adjacent non-cancerous tissues***	Fang Han et al (2021)	10.1007/s00405-021-06789-3	**3**
