# Supplementary material for: Ferroptosis-Related Gene Signatures: Prognostic Role in HPV-Positive Oropharyngeal Squamous Cell Carcinoma
Source: Cancers (Basel). 2025 Feb 5;17(3):530. doi: 10.3390/cancers17030530 (PMC11817470; doi:10.3390/cancers17030530)
Supplement: Supplementary file 1 [file cancers-17-00530-s001.zip › TableS5.pdf]

	FER3	FER6	FER11	FER12
**Imatinib**	-0.37	-0.42	-0.24	-0.5
**Bexarotene**	-0.39	-0.41	-0.22	-0.43
**Cyclopamine**	-0.33	-0.33	-0.4	-0.38
**Dasatinib**	-0.24	-0.31	-0.1	-0.3
**Lapatinib**	-0.25	-0.03	-0.35	-0.29
**Pazopanib**	-0.26	-0.33	-0.29	-0.29
**Docetaxel**	-0.2	-0.23	-0.32	-0.13
**Erlotinib**	-0.1	0.16	-0.12	0.05
**Paclitaxel**	-0.01	0.15	-0.1	0.05
**Cisplatin**	0.05	-0.04	-0.13	0.1
**Bleomycin**	0.2	0.1	-0.04	0.11
**Doxorubicin**	0.2	0.15	-0.16	0.19

Supplementary Table S5: Correlation values between ferroptosis signatures and drugs in Metanalysis-HPV267 dataset.
