# Supplementary material for: Ferroptosis-Related Gene Signatures: Prognostic Role in HPV-Positive Oropharyngeal Squamous Cell Carcinoma
Source: Cancers (Basel). 2025 Feb 5;17(3):530. doi: 10.3390/cancers17030530 (PMC11817470; doi:10.3390/cancers17030530)
Supplement: Supplementary file 1 [file cancers-17-00530-s001.zip › TableS6.pdf]

	FER3	FER4	FER6	FER12
**Cyclopamine**	-0.52	-0.54	-0.55	-0.27
**Docetaxel**	-0.49	-0.48	-0.53	-0.26
**Pazopanib**	-0.45	-0.42	-0.47	-0.19
**Cisplatin**	-0.39	-0.3	-0.43	-0.13
**Dasatinib**	-0.41	-0.51	-0.41	-0.3
**Bleomycin**	-0.4	-0.3	-0.4	-0.1
**Bexarotene**	-0.36	-0.42	-0.32	-0.34
**Doxorubicin**	-0.31	-0.22	-0.32	-0.027
**Erlotinib**	-0.31	-0.34	-0.27	-0.14
**Paclitaxel**	-0.26	-0.32	-0.25	-0.16
**Lapatinib**	-0.31	-0.41	-0.16	-0.33
**Imatinib**	-0.0063	-0.09	0.12	-0.13

Supplementary Table S6: Correlation values between ferroptosis signatures and drugs in BD2-HPV286 dataset.
